# Developing shortcut algorithms for value-based pricing of pharmaceuticals

**DOI:** 10.1186/s13561-026-00757-5

**Published:** 2026-03-18

**Authors:** Afschin Gandjour

**Affiliations:** Frankfurt School of Finance & Management, Adickesallee 32-34, Frankfurt am Main, 60322 Germany

**Keywords:** Value-based pricing, Pharmaceuticals, Health care, Modeling, Extrapolation

## Abstract

Value-based pricing (VBP) involves regulating the reimbursement or pricing of pharmaceuticals based on their therapeutic value. We adopt VBP as the policy context for this study. Unlike alternative approaches, VBP depends on an explicit relationship between price and incremental health outcomes and costs. This dependence makes VBP decisions especially sensitive to uncertainty about downstream costs and outcomes. A central question is whether—and how far—these components must be modeled beyond the trial period. A key gap is that the literature provides limited, generalizable conditions for when extrapolation is necessary for pricing conclusions versus when trial-period evidence is sufficient. This paper demonstrates the conditions under which it is possible to forgo modeling downstream costs beyond the clinical trial period for VBP purposes. It provides a series of specific mathematical proofs to support this approach. The proposed shortcuts reduce the burden and time required to produce evaluations. Furthermore, the shortcuts validate the robustness of decision models by allowing modelers to test whether applying these assumptions yields consistent results, enhancing the reliability of VBP methodologies.

## Introduction

Value-based pricing (VBP) is gaining traction across the European Union (EU) as a method for determining the reimbursement or pricing of pharmaceuticals based on their therapeutic value [24]. Countries like the Netherlands, Norway, Sweden, and the UK utilize formal economic evaluations (cost-effectiveness analysis, CEA) to guide their VBP strategies. Similarly, Belgium, France, and Germany categorize new products to assess added therapeutic benefits and justify pricing premiums for innovative medicines. Italy employs an innovation rating system in price negotiations, supplemented by performance-based agreements. Additionally, Denmark and Spain incorporate various elements of VBP into their health care decision-making processes.

Central to VBP based on a CEA is the use of a cost-effectiveness threshold. This ensures that drug prices align more closely with the actual therapeutic benefits they deliver. It is important to note that VBP in the EU is still evolving, with individual countries adopting elements of VBP within their existing frameworks.

In practice, implementing VBP requires a health technology assessment (HTA) evidence base that links price to incremental costs and health outcomes over an appropriate time horizon. Because pivotal trials rarely observe lifetime effects, this often necessitates decision-analytic modeling and extrapolation beyond the trial period [9]. A major driver of modeling burden—and of disagreement in HTA practice—has been the need to extrapolate outcomes beyond the end of clinical trials, particularly for survival. Methodological guidance and applied reviews underscore that extrapolation is often necessary to estimate lifetime benefits and that different plausible survival models can yield materially different incremental cost-effectiveness ratios (ICERs), making extrapolation both consequential and resource-intensive [18]. This methodological evolution has been accompanied by the development of detailed agency-oriented technical guidance, such as the NICE Decision Support Unit (DSU) technical support documents on survival analysis/extrapolation and later flexible survival modeling approaches, reflecting the increasing sophistication expected in contemporary submissions [17].

Capacity constraints in terms of producing health technology assessments [2] may impede the VBP of drugs, however. Such labor constraints were documented early on for several Central and Eastern European settings, where limited analytic staff and tight timelines made it difficult to generate the evidence required for model-based value assessments. Since then, HTA activity in Europe has continued to expand and professionalize, and EU-level cooperation has intensified—culminating in the Health Technology Assessment (HTA) Regulation (EU) 2021/2282, which establishes a permanent framework for EU joint clinical assessments and has been applicable since 12 January 2025 [11]. Importantly, the policy response to persistent capacity heterogeneity has not been to assume the problem has disappeared, but rather to invest in capacity building and work-sharing. For example, the European Commission explicitly links the HTA Regulation to supporting voluntary cooperation and capacity building of national HTA systems, and has established a dedicated HTAR Capacity Building Programme [10].

Against this backdrop, capacity constraints may still be a key reason why some countries hesitate to introduce—or fully operationalize—VBP approaches that depend on timely, model-intensive cost-effectiveness and HTA production. A justifiable reduction of complex modeling—such as Markov modeling or discrete-event simulation—to a minimum could decrease the burden and time required to produce evaluations, thus facilitating the introduction of VBP in these countries. Similarly, shortcut algorithms or efficient computational methods have been proposed in the area of priority-setting in research, specifically for calculating the value of information [2, 6, 20, 25, 28].

The purpose of this paper is to identify conditions under which VBP of new drugs—narrowly defined as pricing determined through economic evaluations— does not require additional decision-analytic modelling of downstream costs (e.g., cost offsets and life-extension costs) beyond the trial period. For the trial period, an economic evaluation can be conducted either by collecting cost data alongside clinical data (known as ‘piggyback’ economic evaluation) or through decision modeling. Both approaches are deemed acceptable for applying the shortcuts proposed in this paper. As will be explained, these shortcuts are applicable when at least one drug is already on the market, and its price has been set using VBP. This scenario is becoming more common as several therapeutic areas, including multiple myeloma, stroke prevention in atrial fibrillation, and hepatitis C, have seen successive introductions of innovative drugs.

It is important to emphasize that this paper does not aim to refine VBP with different assumptions but to propose feasible shortcuts based on commonly accepted assumptions. Reducing data requirements helps avoid complex modeling, lessens the burden and time required for evaluations, and decreases uncertainty in determining reimbursement prices. This uncertainty often stems from two sources: (i) the unit prices and quantities of resources consumed associated with downstream costs, and (ii) the extrapolation of the magnitude of clinical events into the future.

This paper contributes to the VBP/HTA literature in three ways. First, it derives and proves sufficient conditions under which post-trial downstream components—such as savings from avoided events and survival-related costs—can be omitted without changing the implied value-based price, thereby reducing data and modeling requirements within a value-based pricing framework. Second, it operationalizes these results as a decision procedure that indicates when trial-period evidence is sufficient for pricing and when extrapolation is decision-relevant, addressing a gap in the literature that has focused more on how to extrapolate than on when extrapolation is necessary for the pricing decision. Third, it shows how the proposed shortcuts can serve as transparent internal validation and robustness checks for complex decision models.

Because this study is methodological and derives sufficient conditions analytically, validation focuses on verification of derivations and internal consistency rather than external prediction. Analysts can implement the shortcut assumptions within their model and verify that the implied price (or the incremental impact of downstream components) behaves as predicted. Any discrepancy indicates that model conclusions may be driven by structural choices or implementation details that warrant scrutiny, thereby strengthening confidence in VBP evaluations.

## Methods

Assume there are two interventions: a new drug D, and the next most effective intervention B. Both interventions affect $m$ clinical outcomes/events (CEs). Suppose it is possible to aggregate these $m$ CEs by assigning each CE a severity weight $w$. This results in an index or composite outcome. Weights should be determined empirically, for example, through preference-based questionnaires, conjoint analysis, or analytic hierarchy process.

In this paper, the trial period refers to the observation window covered by the comparative clinical evidence (typically the pivotal randomized trial) used to estimate incremental CEs and costs for $\mathrm{D}$ vs $\mathrm{B}$—i.e., from randomization to the end of follow-up in the base-case trial-period evaluation. For this period, the economic evaluation can be conducted either as a within-trial (“piggyback”) evaluation collecting resource use/cost data alongside the trial, or via a decision model calibrated to reproduce trial-period effects; both approaches are acceptable for applying the proposed shortcuts. The trial period affects the level of the trial-period ICER (and thus the initial value-based price) because it determines the observed incremental clinical outcomes and costs for $\mathrm{D}$ vs $\mathrm{B}$ during follow-up. However, the purpose of the shortcuts is to determine whether extending the analysis beyond this observed period changes the implied value-based price; under the conditions in the propositions, post-trial savings and/or survival costs cancel out, and thus are neutralized regardless of the exact trial length.

We calculate the incremental cost-effectiveness ratio (ICER) of drug D vs intervention B as follows [13]:

1 $$\mathrm{ICER}_{\text{D vs B}}=\frac{\Delta {C}_{\mathrm{drug},\text{ D vs B}}}{\sum_{i=1}^{m}\Delta {CE}_{\text{D vs B},i}\times {w}_{i}}+\frac{\sum_{i=1}^{m}\Delta {CE}_{D vs B,i}\times \left(1-{\alpha }_{i}\right)\times {C}_{\mathrm{CE},i}}{\sum_{i=1}^{m}\Delta {CE}_{\text{D vs B},i}\times {w}_{i}}+ \frac{\sum_{i=1}^{m}\Delta {CE}_{\text{D vs B},i}\times \alpha \times {C}_{\mathrm{s},i}}{\sum_{i=1}^{m}\Delta {CE}_{\text{D vs B},i}\times {w}_{i}},$$

where $C$ denotes costs; $\Delta {C}_{drug}$ captures drug-related costs (e.g., costs of managing adverse events and drug-related services such as counseling, monitoring, and testing); ${C}_{\mathrm{CE},i}$ denotes the unit cost associated with a CE of type $i$ (so that avoided CEs generate savings; ${\alpha }_{i}$ is the probability that a CE of type $i$ is fatal; ${C}_{s,i}$ is the survival (life-extension) cost associated with a fatal CE of type $i$; and $i$ indexes the $m$ CEs considered in the analysis (see Table 1 for notation).

**Table 1:** Notation (ordered by appearance)

Symbol	Type	Definition	Units/notes	Used in
Indices and sets				
$D$	Intervention	New drug	—	Setup; Eq. (1)
$B$	Intervention	Comparator (next most effective alternative)	—	Setup; Eq. (1)
$i = 1,\dots ,m$	Index/set	Index for clinical events/endpoints (CEs);$m$total CEs	*i* indexes CEs	Equation (1)
$CE_{i}$	Outcome category	Clinical event/endpoint *i*	—	Equation (1)
Core outcomes and weights				
$w_{i}$	Parameter	Severity weight for CE *i* in composite outcome	Elicited/estimated (preferences, etc.)	Denominator; Eq. (1)
$\Delta {CE}_{\text{D vs B},i}$	Variable	Incremental number of CE *i* for D vs B over the analysis period	Events avoided	Equation (1); later derivations
Costs in the ICER numerator				
$C_{\mathrm{D}}$	Variable	Total cost under D	Discounted present value	Equation (1)
$C_{\mathrm{B}}$	Variable	Total cost under B	Discounted present value	Equation (1)
$\Delta {C}_{\mathrm{drug},\text{ D vs B}}$	Variable	Incremental drug (and drug-related) costs of D vs B	Discounted present value	Equation (1)
$\Delta {p}_{\mathrm{D} vs \mathrm{B}}$	Variable	Incremental acquisition price of drug D vs B	Discounted present value	Equation (18)
$\Delta {C}_{nonprice,\mathrm{D} vs \mathrm{B}}$	Variable	Incremental non-price drug-related costs of D vs B	Discounted present value	Equation (18)
$C_{\mathrm{CE},i}$	Parameter	Unit cost associated with CE *i* (morbidity-related)	Savings per CE avoided	Equation (1)
$\alpha_{i}$	Parameter	Proportion of CE *i* that is fatal	0 ≤${\alpha }_{i}$≤ 1	Equation (1); assumptions
${C}_{\mathrm{s},i}$	Parameter	Survival (life-extension) costs associated with fatal CE *i*	Costs per avoided death	Equation (1)
Additional inputs introduced later (propositions)				
$Inc_{i}$	Parameter	Incidence/baseline risk of CE *i*		Propositions
$RRR_{i}$	Parameter	Relative risk reduction for CE *i*		Propositions
$k$	Index/horizon	Number of periods beyond the trial/time index		Propositions on extrapolation

Note that ${C}_\mathrm{drug}$ includes both the acquisition price and other drug-related (non-price) costs. When deriving a value-based price, we separate ${C}_\mathrm{drug}$ into a price component $p$ and a residual non-price component. Furthermore, costs and CEs are discounted to express them in present value terms, thus accounting for the timing of events. The equation assumes that the value of ${C}_{\mathrm{CE}}$ and ${C}_{\mathrm{s}}$ is independent of the timing of CEs, that is, does not change over time. We will relax this assumption in the shortcuts described below.

For each CE $i$, we express the incremental number of events avoided during the trial period, $\Delta C{E}_{\mathrm{D} vs \mathrm{B},i}$, as a function of baseline incidence and treatment effect. Specifically, the number of avoided events can be written as the product of an incidence term and a relative risk reduction term, i.e., $\Delta C{E}_{\mathrm{D} vs \mathrm{B},i}\propto In{c}_{i}\times RR{R}_{i}$. This representation is used in the proofs for multiple endpoints and makes explicit that RRR enters the ICER in Eq. (1) only through $\Delta C{E}_{\mathrm{D} vs \mathrm{B},i}$. As a result, changes in RRR scale both the weighted health benefit denominator and the event-driven cost components (savings and survival costs) through the number of avoided events.

Because RRR enters only via $\Delta C{E}_{\mathrm{D} vs \mathrm{B},i}$, standard uncertainty/sensitivity analysis for the RRR remains relevant in any trial-period (within-trial) economic evaluation. In addition, robustness of the proposed shortcuts should be assessed by examining whether their key structural assumptions about treatment effects hold (e.g., RRR being constant over time or changing proportionally at the same rate across comparisons/endpoints). When these conditions hold, the pricing conclusion regarding whether extrapolation is needed is invariant to the specific RRR level; when they are violated (e.g., differential waning), the shortcuts may no longer apply and full extrapolation is required.

According to the second term of Eq. 1, savings from avoiding non-fatal CEs (i.e., reducing morbidity) are calculated by multiplying the resources used to treat CEs by the corresponding unit cost. Put simply, the number of CEs avoided is multiplied by the average cost of treating a CE, following traditional cost-effectiveness analysis norms (e.g., [19, 23]). The calculation assumes approximate linearity in costs and outcomes (proportional scaling and no important indivisibilities), so that expected savings can be expressed as avoided events multiplied by a unit cost [3, 4, 29].

While life-extension costs are somewhat diminished by considering end-of-life costs (which decrease with age) [12], they are not considered here. Typically, cost-effectiveness analysis implicitly assumes that end-of-life costs are the same in both arms and thus cancel out.

Equation 1 forms the basis for the shortcuts that will be described in the following sections. Using quality-adjusted life years (QALYs) instead of CEs would not change results of our analysis and would require the same assumptions. This is because the relationship between QALYs gained and CEs avoided at a given age and comorbidity level is linear. Each CE avoided (at a given age and comorbidity level) yields an equal amount of gained QALYs (see Holtgrave and Qualls [16] for an example). The advantage of modeling CEs instead of QALYs is that it simplifies our analysis, offering a more parsimonious approach because it avoids an extra modeling effort to extrapolate gains in QALYs from avoided CEs. Moreover, this simplification allows for an analytical solution (see, e.g., the proof of proposition 1). We will revisit the use of QALYs in the final sections of the paper.

## Results

In this section, we describe eight propositions that establish the conditions under which VBP does not require modeling downstream costs beyond the trial period. For some of these propositions, the long-term price of drug D (beyond the trial period) aligns with the price determined during the trial period, eliminating the need for further modeling, including the modeling of health benefits, to determine the long-term price of a new drug. The propositions and their assumptions are summarized in Tables 2 and 3, illustrating a systematic treatment of the topic. Although the proposed shortcuts use another drug as the comparator, they remain applicable regardless of comparator type, which may include other medical technologies. All propositions are applicable to primary and secondary prevention, which involves avoiding initial and recurrent CEs, respectively.

**Table 2:** Sufficient conditions for excluding survival costs in cost-effectiveness analyses with multiple endpoints (indicated by a plus sign without brackets)

	Probabilities of death		Weights of CEs		RRR		Incidence	
Proposition no	Equal	Constant ratio over time	Equal	Constant ratio over time	Equal	Constant ratio over time	Equal	Constant ratio over time
2	+				+			
3		(+)		(+)		(+)		(+)
4	+		+					

A (+) indicates that while survival costs cannot be entirely ruled out, their change is consistent across both treatment comparisons

RRR Relative risk reduction

CE Clinical event

**Table 3:** Sufficient conditions for excluding savings in cost-effectiveness analyses with multiple endpoints (indicated by a plus sign without brackets)

	Probabilities of death		Weights of CEs		RRR		Incidence		Costs of CEs	
Proposition no	Equal	Constant ratio over time	Equal	Constant ratio over time	Equal	Constant ratio over time	Equal	Constant ratio over time	Equal	Constant ratio over time
5	+				+					
6		(+)		(+)		(+)		(+)		(+)
7	+		+						+	

A (+) indicates that while savings cannot be entirely ruled out, their change is consistent across both treatment comparisons

RRR Relative risk reduction

CE Clinical event

To derive the pricing conditions, we adopt a three-intervention framework consisting of a new drug $\mathrm{D}$, its direct comparator $\mathrm{B}$, and a reference intervention $\mathrm{A}$. While the primary comparison concerns $\mathrm{D}$ versus $\mathrm{B}$, consistency of value-based pricing requires that the implied value-based price condition for $\mathrm{D}$ vs $\mathrm{B}$ is compatible with that between $\mathrm{B}$ and $\mathrm{A}$, assuming the same threshold $\lambda$ applies to both comparisons (so each ICER is set equal to $\lambda$ under VBP). Embedding the analysis in this $\mathrm{D}\to \mathrm{B}\to \mathrm{A}$ sequence ensures internal consistency of the pricing condition across adjacent comparisons.

The first proposition, previously presented in the context of internal validation of cost-effectiveness models with multiple interventions [13], is now applied in the VBP context to demonstrate its broader applicability. It addresses the scenario where a new drug and its comparator affect only one CE. Propositions 2 to 7 concern scenarios where multiple CEs are affected: Propositions 2 to 4 focus on life-extension costs from avoiding multiple CEs, with concurrent savings excluded for simplicity. Propositions 5 to 7, in contrast, deal with savings from avoiding multiple CEs, excluding life-extension costs.

Propositions involving a constant ratio (or constant relative change) of model inputs over time cover cases where model inputs do not vary across endpoints (e.g., fatality across endpoints). Thus, when model inputs are uniform across endpoints, any change over time impacts all endpoints equally. Following the discussion of these propositions, we will present empirical evidence supporting the underlying conditions.

### Proposition 1

When a new drug and its comparator affect only one CE, savings and survival costs are neutralized in the analysis.

#### Proof

Consider the trial period. Assume that the new drug D and its comparator B are priced such that their ICER matches the threshold willingness to pay, and assume this threshold is the same for both interventions. For a single CE, based on Eq. 1, we derive the following condition:

2 $$\begin{array}{c}\frac{\Delta {C}_{\mathrm{drug},\text{D vs B}}+\Delta {CE}_{\text{D vs B}}\times \left(1-\alpha \right)\times {C}_{\mathrm{CE}}+\Delta {CE}_{\text{D vs B}}\times \alpha \times {C}_{\mathrm{s}}}{\Delta {CE}_{\text{D vs B}}}\\ =\frac{\Delta {C}_{\mathrm{drug},\text{ B vs A}}+\Delta {CE}_{\text{B vs A}}\times \left(1-\alpha \right)\times {C}_{\mathrm{CE}}+\Delta {CE}_{\text{B vs A}}\times \alpha \times {C}_{\mathrm{s}}}{\Delta {CE}_{\text{B vs A}}}\end{array}$$

The equation assumes that $\alpha$, the proportion of fatal outcomes, is consistent across both comparisons. If trial data indicate variability in $\alpha$, further investigation is required to understand the reasons behind this discrepancy. Potential explanations could include differing types of clinical outcomes, such as variations in the severity of type of myocardial infarction, or differing fatality rates. These scenarios align with the multiple outcomes described in Propositions 2 to 7. Additionally, differences in $\alpha$ may arise from variations in concomitant therapies, possibly due to trials being conducted at different times or in different countries. In such cases, analysts must decide whether to apply the same fatality rate across their analyses.

When the condition of equal $\alpha$s applies, it is evident that savings and life-extension costs cancel out from Eq. 1, as shown below:

3 $$\frac{\Delta {C}_{\mathrm{drug},\text{ D vs B}}}{\Delta {CE}_{\text{D vs B}}}=\frac{\Delta {C}_{\mathrm{drug},\text{ B vs A}}}{\Delta {CE}_{\text{B vs A}}}$$

While costs of survival are influenced by repetitive CEs beyond the trial period (leading to additional survival costs and benefits by avoiding them), they still cancel out. This occurs because changes in survival costs and benefits across treatments in the subsequent time period are entirely caused by avoided CEs in that period. Since the relationship of survival costs and benefits to CEs avoided remains the same for both treatment comparisons, survival costs and benefits cancel out again. Moreover, if the relative risk reduction (RRR) does not change over time or changes at the same rate for both comparators, the ratios of $\Delta {CE}_{\text{D vs B}}$ and $\Delta {CE}_{\text{B vs A}}$ in Eq. 3 remain constant, and so does the ratio in incremental drug costs. Given that the price of drug B is fixed, the price of drug D will also remain unchanged.

### Proposition 2

When a new drug and its comparator prevent several CEs that are partially fatal, life-extension costs cancel out under the following conditions: each endpoint is associated with the same probability of death, the RRR by endpoint is the same, and the RRR stays constant over time.

#### Proof

First, consider the trial period. Using Eq. (1) for multiple endpoints and imposing the VBP condition (equal threshold) yields:

4 $$\begin{array}{c}\frac{\Delta {C}_{\mathrm{drug},\text{ D vs B}}+\sum_{i=1}^{m}\Delta {CE}_{\text{D vs B},i}\times \alpha \times {C}_{\mathrm{s}}}{\sum_{i=1}^{m}\Delta {CE}_{\text{D vs B},i}\times {w}_{i}}\\ =\frac{\Delta {C}_{\mathrm{drug},\text{ B vs A}}+\sum_{i=1}^{m}\Delta {CE}_{\text{B vs A},i}\times \alpha \times {C}_{\mathrm{s}}}{\sum_{i=1}^{m}\Delta {CE}_{\text{B vs A},i}\times {w}_{i}}\end{array}$$

Considering that the number of CEs avoided is a product of incidence ($Inc$) and relative risk reduction ($RRR$), we derive:

5 $$\begin{array}{c}\frac{\Delta {C}_{\mathrm{drug},\text{ D vs B}}+\sum_{i=1}^{m}{{Inc}_{i}\times RRR}_{\text{D vs B} }\times \alpha \times {C}_{\mathrm{s}}}{\sum_{i=1}^{m}{{Inc}_{i}\times RRR}_{\text{D vs B}}\times {w}_{i}}\\ =\frac{\Delta {C}_{\mathrm{drug},\text{B vs A}}+\sum_{i=1}^{m}{{Inc}_{i}\times RRR}_{\text{B vs A}}\times \alpha \times {C}_{\mathrm{s}}}{\sum_{i=1}^{m}{{Inc}_{i}\times RRR}_{\text{B vs A}}\times {w}_{i}}\end{array}$$

Under the proposition’s assumption that the $RRR$ is identical across endpoints (i.e., a common scalar), it factors out of both the numerator and denominator of the survival-cost component, yielding:

6 $$\begin{array}{c}\frac{\Delta {C}_{\mathrm{drug},\text{ D vs B}}}{\sum_{i=1}^{m}{{Inc}_{i}\times RRR}_{\text{D vs B}}\times {w}_{i}}+\frac{\alpha \times {C}_{\mathrm{s}}\times \sum_{i=1}^{m}{Inc}_{i}}{\sum_{i=1}^{m}{Inc}_{i}\times {w}_{i}}\\ =\frac{\Delta {C}_{\mathrm{drug},\text{ B vs A}}}{\sum_{i=1}^{m}{{Inc}_{i}\times RRR}_{\text{B vs A}}\times {w}_{i}}+\frac{\alpha \times {C}_{\mathrm{s}}\times \sum_{i=1}^{m}{Inc}_{i}}{\sum_{i=1}^{m}{Inc}_{i}\times {w}_{i}}\end{array}$$

Since all parameters in the second terms do not depend on the interventions in question, they are identical and therefore cancel out, eliminating life-extension costs. Furthermore, as the RRR does not change over time, the price of drug D will also not change.

### Proposition 3

When a new drug and its comparator prevent several CEs, changes in life-extension costs do not affect the implied value-based price (because they change proportionally in both treatment comparisons and thus cancel out in the pricing condition) if the following conditions are satisfied: CEs are associated with a constant ratio (or constant relative change) of i) probabilities of death across endpoints, ii) RRRs across endpoints, iii) incidence rates across endpoints, and iv) weights of CEs.

#### Proof

Consider the trial period. Starting from Eq. 4 and considering that the number of CEs avoided is a product of incidence ($Inc$) and relative risk reduction ($RRR$), we derive:

7 $$\begin{array}{c}\frac{\Delta {C}_{\mathrm{drug},\text{ D vs B}}+\sum_{i=1}^{m}{{Inc}_{i}\times RRR}_{\text{D vs B},i}\times {a}_{i}\times {C}_{\mathrm{s}}}{\sum_{i=1}^{m}{{Inc}_{i}\times RRR}_{\text{D vs B},i}\times {w}_{i}}\\ =\frac{\Delta {C}_{\mathrm{drug},\text{ B vs A}}+\sum_{i=1}^{m}{{Inc}_{i}\times RRR}_{\text{B vs A},i}\times {a}_{i}\times {C}_{\mathrm{s}}}{\sum_{i=1}^{m}{{Inc}_{i}\times RRR}_{\text{B vs A},i}\times {w}_{i}}\end{array}$$

Extrapolating beyond the trial period, we introduce constants ${k}_{\mathrm{RRR}}$*, *${k}_{\mathrm{Inc}}$*,* and ${k}_{a}$ to ensure that RRR for each CE, the incidence of each CE, and the probability of dying from each CE change uniformly across all endpoints over *k* periods:

8 $$\begin{array}{c}\frac{\Delta {C}_{\mathrm{drug},\text{ D vs B}}+{{k}_{\mathrm{Inc}}\times k}_{\mathrm{RRR}}\times {k}_{a}\times \sum_{i=1}^{m}{{Inc}_{i}\times RRR}_{\text{D vs B},i}\times {a}_{i}\times {C}_{\mathrm{s}}}{{{k}_{\mathrm{Inc}}\times k}_{\mathrm{RRR}}\times \sum_{i=1}^{m}{{Inc}_{i}\times RRR}_{\text{D vs B},i}\times {w}_{i}}\\ =\frac{\Delta {C}_{\mathrm{drug},\text{ B vs A}}+{{k}_{\mathrm{Inc}}\times k}_{\mathrm{RRR}}\times {k}_{a}\times \sum_{i=1}^{m}{{Inc}_{i}\times RRR}_{\text{B vs A},i}\times {a}_{i}\times {C}_{\mathrm{s}}}{{{k}_{\mathrm{Inc}}\times k}_{\mathrm{RRR}}\times \sum_{i=1}^{m}{{Inc}_{i}\times RRR}_{\text{B vs A},i}\times {w}_{i}}\end{array}$$

Equation (8) shows that post-trial extrapolation enters through the common scaling factor ${k}_\mathrm{Inc}{k}_\mathrm{RRR}$, which captures proportional changes in avoided events (and thus in weighted health benefits) beyond the trial period. In the life-extension cost component, this factor multiplies both the numerator (fatal events avoided) and the denominator (weighted effects) and therefore cancels within each comparison. The factor ${k}_{a}$ does not cancel mechanically. However, under the proportionality assumptions of the proposition, the weighted survival-cost per unit of weighted effect is identical across both comparisons. Consequently, the pricing condition contains the same additive term ${k}_{a}\cdot\Phi$ on both sides, where $\Phi$ denotes the common survival-cost ratio. When the two expressions are equated, this term cancels, and ${k}_{a}$ therefore drops out of the pricing condition. By contrast, ${k}_\mathrm{Inc}{k}_\mathrm{RRR}$ remains in the drug-cost component (the first term) through the scaling of the denominator. Nevertheless, because the same proportional scaling is assumed to apply to both treatment comparisons in Eq. (8), extending the horizon does not induce a differential price adjustment beyond the trial period under the proposition’s conditions. Any price change would require non-proportional changes (e.g., differential time trends in effects, deviations in ${k}_{a}$, or non-proportional changes in endpoint weights ${w}_{i}$ or fatality patterns).

Using Eq. (8), modest departures from proportionality can be parameterized as multiplicative deviations in the scaling factors (i.e., $k$ near 1). Given a chosen pricing tolerance (e.g., requiring $\mid\Delta p\mid$ to remain within ± 5% of the trial-based price), one can solve for the range of $k$ that satisfies this constraint. For small deviations in $k$, the induced price change is approximately linear in $(k-1)$ and is driven by the magnitude of the post-trial component and by departures from common scaling across comparisons/endpoints (in particular, deviations in ${k}_{\alpha }$ and non-proportional changes in endpoint weights or fatality patterns).

If the severity of each CE changes proportionally, the weights assigned to these CEs remain constant and the weighted sum of incident events or CEs remains unchanged. Therefore, a proportional increase in severity does not alter the outcome of the equation, effectively canceling out any impact from such changes. As this principle applies to subsequent proofs, it will not be reiterated.

### Proposition 4

When a new drug and its comparator prevent several CEs, life-extension costs cancel out when each endpoint is associated with i) the same probability of death and ii) the same weight.

#### Proof

First, consider the trial period. From Eq. 1, we obtain:

9 $$\begin{array}{c}\frac{\Delta {C}_{\mathrm{drug},\text{ D vs B}}+\sum_{i=1}^{m}\Delta {CE}_{\text{D vs B},i}\times a\times {C}_{\mathrm{s}}}{\sum_{i=1}^{m}\Delta {CE}_{\text{D vs B},i}}\\ =\frac{\Delta {C}_{\mathrm{drug},\text{ B vs A}}+\sum_{i=1}^{m}\Delta {CE}_{\text{B vs A},i}\times a\times {C}_{\mathrm{s}}}{\sum_{i=1}^{m}\Delta {CE}_{\text{B vs A},i}}\end{array}$$

When the weights of CEs are the same, benefits from reducing each CE are simply summed to calculate total health benefit. Moreover, if each endpoint has the same probability of death ($a$), the life-extension cost component is identical on both sides and therefore cancels. Similar to Proposition 1, if RRR does not change over time or changes at the same rate for both comparators, the price of drug D remains stable.

### Proposition 5

When a new drug and its comparator prevent several CEs, savings cancel out when each endpoint is associated with the same probability of death, and the RRR by endpoint is the same and remains constant over time.

#### Proof

First, consider the trial period. Using Eq. 1, we derive:

10 $$\begin{array}{c}\frac{\Delta {C}_{\mathrm{drug},\text{ D vs B}}+\sum_{i=1}^{m}\Delta {CE}_{\text{D vs B},i}\times \left(1-a\right)\times {C}_{\mathrm{CE},i}}{\sum_{i=1}^{m}\Delta {CE}_{\text{D vs B},i}\times {w}_{i}}\\ =\frac{\Delta {C}_{\mathrm{drug},\text{ B vs A}}+\sum_{i=1}^{m}\Delta {CE}_{\text{B vs A},i}\times \left(1-a\right)\times {C}_{\mathrm{CE},i}}{\sum_{i=1}^{m}\Delta {CE}_{\text{B vs A},i}\times {w}_{i}}\end{array}$$

Note that while the proposition focuses on savings, probabilities of death are still relevant because savings are realized only from avoiding non-fatal CEs. As this reasoning applies to subsequent propositions, it will not be reiterated.

Considering that the number of CEs avoided is a product of incidence ($Inc$) and relative risk reduction ($RRR$), we obtain:

11 $$\begin{array}{c}\frac{\Delta {C}_{\mathrm{drug},\text{ D vs B}}+\sum_{i=1}^{m}{{Inc}_{i}\times RRR}_{\text{D vs B} }\times \left(1-a\right)\times {C}_{\mathrm{CE},i}}{\sum_{i=1}^{m}{{Inc}_{i}\times RRR}_{\text{D vs B} }\times {w}_{i}}\\ =\frac{\Delta {C}_{\mathrm{drug},\text{ B vs A}}+\sum_{i=1}^{m}{{Inc}_{i}\times RRR}_{\text{B vs A}}\times \left(1-a\right)\times {C}_{\mathrm{CE},i}}{\sum_{i=1}^{m}{{Inc}_{i}\times RRR}_{\text{B vs A}}\times {w}_{i}}\end{array}$$

It becomes evident that the $RRR$ factor cancels out from the second terms, simplifying to:

12 $$\begin{array}{c}\frac{\Delta {C}_{\mathrm{drug},\text{ D vs B}}}{\sum_{i=1}^{m}{{Inc}_{i}\times RRR}_{\text{D vs B}}\times {w}_{i}}+\frac{\sum_{i=1}^{m}{Inc}_{i}\times \left(1-a\right)\times {C}_{\mathrm{CE},i}}{\sum_{i=1}^{m}{Inc}_{i}\times {w}_{i}}\\ =\frac{\Delta {C}_{\mathrm{drug},\text{B vs A}}}{\sum_{i=1}^{m}{{Inc}_{i}\times RRR}_{\text{B vs A}}\times {w}_{i}}+\frac{\sum_{i=1}^{m}{Inc}_{i}\times \left(1-a\right)\times {C}_{\mathrm{CE},i}}{\sum_{i=1}^{m}{Inc}_{i}\times {w}_{i}}\end{array}$$

The second terms cancel out completely as all parameters are independent of the interventions in question. As with Proposition 2, since the RRR does not change over time, the price of drug D remains unchanged.

### Proposition 6

When a new drug and its comparator affect several CEs to the same degree, a change in savings over time cancels out when the following conditions are satisfied: CEs are associated with a constant ratio of i) probabilities of death across endpoints, ii) RRRs across endpoints, iii) incidence rates across endpoints, iv) weights of CEs, and v) costs of CEs.

#### Proof

First, consider the trial period. From Eq. 1, we obtain:

13 $$\begin{array}{c}\frac{\Delta {C}_{\mathrm{drug},\text{ D vs B}}+\sum_{i=1}^{m}{\Delta CE}_{\text{D vs B},i }\times \left(1-{a}_{i}\right)\times {C}_{\mathrm{CE},i}}{\sum_{i=1}^{m}{\Delta CE}_{\text{D vs B},i }\times {w}_{i}}\\ =\frac{\Delta {C}_{\mathrm{drug},\text{ B vs A}}+\sum_{i=1}^{m}{\Delta CE}_{\text{B vs A},i }\times \left(1-{a}_{i}\right)\times {C}_{\mathrm{CE},i}}{\sum_{i=1}^{m}\Delta {CE}_{\text{B vs A},i }\times {w}_{i}}\end{array}$$

Given that the number of CEs avoided is a product of incidence ($Inc$) and relative risk reduction ($RRR$), we derive:

14 $$\begin{array}{c}\frac{\Delta {C}_{\mathrm{drug},\text{ D vs B}}+\sum_{i=1}^{m}{{Inc}_{i}\times RRR}_{\text{D vs B},i }\times \left(1-{a}_{i}\right)\times {C}_{\mathrm{CE},i}}{\sum_{i=1}^{m}{{Inc}_{i}\times RRR}_{\text{D vs B},i }\times {w}_{i}}\\ =\frac{\Delta {C}_{\mathrm{drug},\text{ B vs A}}+\sum_{i=1}^{m}{{Inc}_{i}\times RRR}_{\text{B vs A},i }\times \left(1-{a}_{i}\right)\times {C}_{\mathrm{CE},i}}{\sum_{i=1}^{m}{{Inc}_{i}\times RRR}_{\text{B vs A},i }\times {w}_{i}}\end{array}$$

When extrapolating beyond the trial period, we introduce constants ${k}_{\mathrm{RRR}}$*, *${k}_{\mathrm{Inc}}$*,* ${k}_{a}$, and ${k}_{\mathrm{C}}$ to ensure that RRR, incidence, probability of dying from a CE, and costs of CEs change uniformly across all endpoints over $k$ periods:

15 $$\begin{array}{c}\frac{\Delta C_{\text{drug, D vs B}}+k_{\mathrm{Inc}}\times k_\mathrm{RRR}\times k_\mathrm{C}\times\sum_{i=1}^m{{Inc}_i\times RRR}_{\text{D vs B},i}\times\left(1-k_{\alpha}\times\alpha_{i}\right)\times C_{\mathrm{CE},i}}{k_{\mathrm{Inc}}\times k_\mathrm{RRR}\times\sum_{i=1}^m{{Inc}_i\times RRR}_{\text{D vs B},i}\times w_i}\\=\frac{\Delta C_{\text{drug, B vs A}}+{k_\mathrm{Inc}\times k}_\mathrm{RRR}\times k_\mathrm{C}\times\sum_{i=1}^m{{Inc}_i\times RRR}_{\text{B vs A},i}\times\left(1-k_\alpha\times\alpha_i\right)\times C_{\mathrm{CE},i}}{k_{\mathrm{Inc}}\times k_{\mathrm{RRR}}\times\sum_{i=1}^m{{Inc}_i\times RRR}_{\text{B vs A},i}\times w_i}\end{array}$$

The common factor ${k}_\mathrm{Inc}{k}_\mathrm{RRR}$cancels from the CE-cost component, yielding:

16 $$\begin{array}{c}\frac{\Delta C_{\mathrm{drug},\text{D vs B}}}{{k_\mathrm{Inc}\times k}_\mathrm{RRR}\times\sum_{i=1}^m{\mathrm{Inc}_i\times \mathrm{RRR}}_{\text{D vs B},i}\times w_i}+\frac{k_\mathrm{C}\times\sum_{i=1}^m{\mathrm{Inc}_i\times \mathrm{RRR}}_\text{D vs B}\times C_{\mathrm{CE},i}}{\sum_{i=1}^m{\mathrm{Inc}_i\times \mathrm{RRR}}_{\text{D vs B},i}\times w_i}-\frac{k_\mathrm{C}\times k_a\times\sum_{i=1}^m{\mathrm{Inc}_i\times \mathrm{RRR}}_{\text{D vs B},i}\times\alpha{}_i\times C_{\mathrm{CE},i}}{\sum_{i=1}^m{\mathrm{Inc}_i\times \mathrm{RRR}}_{\text{D vs B},i}\times w_i}\\=\frac{\Delta C_{\mathrm{drug},\text{B vs A}}}{{k_\mathrm{Inc}\times k}_\mathrm{RRR}\times\sum_{i=1}^m{\mathrm{Inc}_i\times \mathrm{RRR}}_{\text{B vs A},i}\times w_i}+\frac{k_\mathrm{C}\times\sum_{i=1}^m{\mathrm{Inc}_i\times \mathrm{RRR}}_\text{B vs A}\times C_{\mathrm{CE},i}}{\sum_{i=1}^m{\mathrm{Inc}_i\times \mathrm{RRR}}_{\text{D vs B},i}\times w_i}-\frac{k_\mathrm{C}\times k_a\times\sum_{i=1}^m{\mathrm{Inc}_i\times \mathrm{RRR}}_{\text{B vs A},i}\times\alpha{}_i\times C_{\mathrm{CE},i}}{\sum_{i=1}^m{\mathrm{Inc}_i\times \mathrm{RRR}}_{\text{D vs B},i}\times w_i}\end{array}$$

Hence, the second terms change only by ${k}_{C}$, while the third terms change only by the product ${k}_{C}{k}_{a}$. Under the proposition’s proportionality assumptions, these scaling factors apply identically in both treatment comparisons, so the change in savings over time is consistent across comparisons. Moreover, because the extrapolation factor ${k}_\mathrm{Inc}{k}_\mathrm{RRR}$ scales the drug-cost component in the same way on both sides, extending the horizon does not by itself induce a differential price adjustment beyond the trial period under these conditions. 

Equation (16) makes clear that the shortcut hinges on proportionality: if the post-trial components are approximately proportional to their trial-period counterparts via common multipliers (e.g., avoided events scaling with ${k}_\mathrm{Inc}{k}_\mathrm{RRR}$, event-cost savings scaling with ${k}_{C}$, and the fatal component scaling with ${k}_{C}{k}_{a}$), then the implied price adjustment from extending the horizon remains small. Deviations in the multipliers scale the terms they multiply proportionally (e.g., $k_{\mathrm{Inc}}\;\mathrm{and}\;k_{\mathrm{RRR}}$ scales the avoided-event aggregate in the denominator), while the resulting change in the ICER (and implied price) is approximately linear in $(k-1)$ for small deviations because the ICER is a ratio and not all components scale. Departures that increase post-trial effects (i.e., larger $k_{\mathrm{Inc}}\;\mathrm{and}\;k_{\mathrm{RRR}}$) tend to increase the allowable VBP price, whereas departures that increase post-trial downstream costs (larger ${k}_{C}$ and/or ${k}_{a}$) tend to reduce it. The magnitude of any price change is therefore driven by (i) how far the $k$-factors depart from unity and (ii) how large the post-trial component is relative to the trial-period component.

Using Eq. (16), modest heterogeneity in post-trial extrapolation can be represented as multiplicative deviations in the constant-ratio factors around unity (i.e., $k$ near 1). Given a pre-specified pricing tolerance (e.g., requiring $\mid\Delta p\mid \le 5\mathrm{\%}$ of the trial-based price), one can then derive the range of admissible deviations in $k$ (e.g., values within $[0.9,1.1]$) that keep the implied price change within this tolerance. When $k$ is close to 1, the parts of the pricing condition that depend on $k$—often through ratio terms such as $1/k$—change almost linearly with $(k-1)$. The resulting price adjustment is small when the post-trial component is small, because only a limited share of the pricing condition is affected by post-trial scaling. Moreover, under the proportionality conditions, additional cancellation within and across comparisons further reduces the residual impact of post-trial extrapolation on the implied price.

### Proposition 7

When a new drug and its comparator affect several CEs to the same degree, savings cancel out when the following conditions are satisfied: i) the same probabilities of death across CEs, ii) uniform weights of CEs, and iii) uniform CE unit costs.

#### Proof

Consider the trial period. From Eq. 1, we derive:

17 $$\begin{array}{c}\frac{\Delta {C}_{\mathrm{drug},\text{ D vs B}}+\sum_{i=1}^{m}{\Delta CE}_{\text{D vs B} }\times \left(1-\alpha \right)\times {C}_{\mathrm{CE}}}{\sum_{i=1}^{m}{\Delta CE}_{\text{D vs B} }}\\ =\frac{\Delta {C}_{\mathrm{drug},\text{ B vs A}}+\sum_{i=1}^{m}{\Delta CE}_{\text{B vs A} }\times \left(1-\alpha \right)\times {C}_{\mathrm{CE}}}{\sum_{i=1}^{m}{\Delta CE}_{\text{B vs A}}}\end{array}$$

The savings components cancel out completely in the equation. Additionally, if the RRR remains constant or changes at the same rate for both comparators over time, the price of drug D will also remain unchanged.

Implication for value-based pricing: If we assume the comparator price ${p}_{\mathrm{B}}$ is fixed and value-based, the value-based price for the new drug $D$ can be obtained by setting ${ICER}_{\mathrm{D} vs \mathrm{B}}=\lambda$ in Eq. (1) and solving for ${p}_{\mathrm{D}}$. This yields

18 $${p}_{\mathrm{D}}={p}_{\mathrm{B}}+\lambda \cdot\Delta {E}_{\mathrm{D} vs \mathrm{B}}-\Delta {C}_{nonprice,\mathrm{D} vs \mathrm{B}}-\Delta {C}_{downstream,\mathrm{D} vs \mathrm{B}}$$

Under Proposition 7, the savings component of $\Delta {C}_{downstream,\mathrm{D} vs \mathrm{B}}$ cancels out; therefore, savings do not affect the implied value-based price. Moreover, if relative risk reduction (RRR) remains constant over time (or changes at the same proportional rate across the relevant comparisons), extending the horizon does not change ${p}_{\mathrm{D}}$. In that case, the value-based price is driven by the incremental health benefit $\Delta {E}_{\mathrm{D} vs \mathrm{B}}$ (and any non-price drug-related cost differences).

### Subcase

When a new drug and its comparator prevent death not only by preventing CEs but also by reducing the probability of dying once a CE has occurred, we consider a subcase of Propositions 2–4. This intervention leads to larger survival benefits but also incurs higher survival costs. For horizon invariance in this subcase, we additionally assume that disease-specific and disease-unrelated survival expenditures evolve proportionally with age (i.e., their ratio remains constant over time). From Eq. 1:

19 $$\begin{aligned}&\frac{\Delta{C}_{\text{drug,D vs B}}+\Sigma_{i=1}^{m}\Delta{C}E_{(\text{D vs B},i)}\times\alpha_{i}\times{C}_{s}+\Sigma_{i=1}^{m}\Delta\alpha_{(\text{D vs B},i)}\times{C}_{\mathrm{s}(\alpha)}}{\Sigma_{i=1}^{m}\Delta{CE}_{\text{D vs B},i}\times\alpha_{i}+\Delta\alpha_{(\text{D vs B},i)}}\\=&\frac{\Delta{C}_{\text{drug,B vs A}}+\Sigma_{i=1}^{m}\Delta{C}E_{(\text{B vs A},i)}\times\alpha_{i}\times{C}_{s}+\Sigma_{i=1}^{m}\Delta\alpha_{(\text{B vs A},i)}\times{C}_{\mathrm{s}(\alpha)}}{\Sigma_{i=1}^{m}\Delta{CE}_{(\text{B vs A},i)}\times\alpha_{i}+\Delta\alpha_{(\text{B vs A},i)}}\end{aligned}$$

The denominator represents avoided deaths. Unlike Propositions 2 to 4, this case includes two distinct components of survival costs: disease-specific survival expenditures and disease-unrelated survival expenditures. In primary prevention (preventing first CEs), individuals are initially disease-free, so disease-related survival costs arise only for those who nonetheless experience a CE and survive due to the reduction in case-fatality (the $\Delta \alpha$ component). Otherwise, the relevant survival costs are disease-unrelated (${C}_{s}$). In contrast, secondary prevention (preventing recurrent CEs or reducing mortality conditional on a CE) incurs disease-related survival costs (${C}_{s(\alpha )}$), as individuals are already affected by the disease. Additional costs from unrelated diseases during an extended lifetime might still need to be considered in either setting.

Now, consider modeling cost-effectiveness beyond the trial period over the time horizon *k*. For primary prevention, horizon invariance requires an additional assumption beyond those in Propositions 2–4: Disease-specific and unrelated expenditures increase at the same rate with age. This condition ensures that survival costs associated with preventing CEs and reducing the probability of dying from a CE increase uniformly with age. Since this parallel change in disease-specific and non-specific expenditures affects both comparisons equally, it does not alter cost-effectiveness beyond the trial period. For secondary prevention, where the focus is on preventing repeated CEs, this assumption is also required if the survival costs for preventing repeated CEs differ from those for reducing the probability of dying from these CEs. Regardless of the prevention type, if case-fatality probabilities remain constant (or change uniformly) and the post-trial evolution of avoided events is proportional across the relevant comparisons (e.g., RRR and incidence do not change differentially between D vs B and B vs A), then extending the horizon does not change the implied price of drug D.

### Illustrative application of the shortcuts

Table 4 summarizes the proposed decision procedure (pseudocode) for determining when extrapolation beyond the trial period is unnecessary for value-based pricing. We demonstrate the procedure with a simple, stylized worked example that contrasts (i) a trial-period (“trial-only”) evaluation with (ii) a baseline full-model evaluation that extends the horizon by including post-trial costs and effects. The example uses two clinical endpoints (one non-fatal and one fatal), aggregates effects using severity weights, and decomposes costs into drug-related costs, event-related costs/savings, and survival-related costs, consistent with Eq. (1). Table 5 reports the worked-example inputs and the corresponding trial-only and extended-horizon ICER expressions and implied trial-based value-based price. In the stylized example, the post-trial downstream increment is chosen for simplicity so that $\Delta {C}_{down}^{P}/\Delta {E}^{P}=\lambda$, ensuring that the implied value-based price is unchanged when extending the horizon. More generally, the shortcuts require only that post-trial components enter symmetrically (or proportionally) in the relevant pricing condition, so that they do not induce a differential price adjustment.

**Table 4:** Pseudocode for the shortcut algorithm

1. Input: Trial-period estimates for $\Delta {C}_{\mathrm{nonprice}}$, $\Delta C{E}_{i}$, ${w}_{i}$, ${C}_{CE,i}$, ${\alpha }_{i}$, ${C}_{s,i}$; comparator price ${p}_{B}$; threshold $\lambda$
2. Compute trial-period ICER components using Eq. (1) and derive the trial-based VBP price ${p}^{(T)}$ by setting $ICE{R}^{(T)}=\lambda$ and solving for ${p}_{D}$
3. Check applicability conditions (per propositions/Tables 2–3): required time-invariance/proportionality relationships (e.g., for event patterns, fatality shares, and—if used—RRR stability/proportional change)
4. If the applicable proposition implies price invariance (i.e., it concludes $p$ is unchanged beyond trial given the RRR condition), set $p={p}^{(T)}$ and stop
5. Else (partial shortcut): report which downstream term(s) can be omitted (e.g., survival costs and/or savings), and compute $p$ using the remaining required terms (which may still require some extrapolation)

**Table 5:** Worked example: trial-only vs full-model ICER and implied VBP price

Quantity	Symbol	Trial period	Post-trial (condition holds)	Total
Inputs				
Avoided non-fatal events	$\Delta C{E}_{1}$	0.10	—	0.10
Avoided fatal events	$\Delta C{E}_{2}$	0.02	—	0.02
Weights	${w}_{1},{w}_{2}$	0.2, 1.0	—	0.2, 1.0
Threshold	$\lambda$	€50,000	—	—
Savings per non-fatal event	${C}_{CE,1}$	− €5,000	—	− €5,000
Survival cost per avoided death	${C}_{s,2}$	€30,000	—	—
Non-price drug-related cost	$\Delta {C}_{nonprice}$	€1,000	—	—
Derived quantities				
Weighted effect	$\Delta E={\sum }_{i}\Delta C{E}_{i}{w}_{i}$	0.04	0.02	0.06
Downstream cost (net)	$\Delta {C}_{down}$	€100	€1,000	€1,100
Total incremental cost	$\Delta {C}_{total}=p+\Delta {C}_{nonprice}+\Delta {C}_{down}$	$p+\mathrm{1,100}$	—	$p+\mathrm{2,100}$
ICER	$ICER=\Delta {C}_{total}/\Delta E$	$(p+\mathrm{1,100})/0.04$	—	$(p+\mathrm{2,100})/0.06$
VBP price solving $ICER=\lambda$	${p}^{(T)}$	€900	—	€900 (unchanged)

1. Downstream cost (trial):$\Delta {C}_{down}^{T}=\Delta C{E}_{1}\cdot {C}_{CE,1}+\Delta C{E}_{2}\cdot {C}_{s,2}=0.10(-\mathrm{5,000})+0.02(\mathrm{30,000})={\in}100$

2. For illustrative purposes, the post-trial downstream increment is chosen so that the overall ICER at the trial-based price remains equal to the threshold when the horizon is extended; this is achieved by imposing$\Delta {C}_{down}^{P}=\lambda \hspace{0.17em}\Delta {E}^{P}$. Here,$\Delta {E}^{P}=0.02$implies$\Delta {C}_{down}^{P}=\mathrm{50,000}\cdot 0.02={\in}1000$

3.$p$ denotes the incremental acquisition price of D vs B; $\Delta {C}_{nonprice}$ captures incremental non-price drug-related costs. Under the construction in (2), extending the horizon leaves the implied VBP price unchanged

To complement the stylized worked example, we provide a brief retrospective illustration from a pharmaceutical setting—sacubitril/valsartan (Entresto) versus valsartan in HFpEF—based on PARAGON-HF (see Table 6 for the parameter inputs used in this illustration). PARAGON-HF reported no material difference in cardiovascular death between arms (hazard ratio 0.95; 95% CI 0.79–1.16), while recurrent heart-failure hospitalizations were lower with sacubitril/valsartan (690 vs 797 total hospitalizations; rate ratio 0.85; 95% CI 0.72–1.00) [27]. This pattern—reduced morbidity without a survival gain—motivates the use of shortcuts that imply horizon-invariance of the value-based price, because extending the horizon does not add survival-related costs when survival is unchanged and the morbidity-related benefits and associated cost offsets remain broadly stable (or change proportionally) over time.

**Table 6:** Parameters for illustrative calculation

Parameter	Symbol	Value
Trial duration	$T$	3 years
Control hospitalization incidence	$Inc$	0.15 events/person-year
Relative reduction in hospitalizations	$RRR$	0.15
Cost per hospitalization	${C}_{\mathrm{CE}}$	€10,000
Annual QALY gain (from fewer hospitalizations)	$\Delta E$	0.04 QALYs/year
Threshold	$\lambda$	€50,000/QALY

Assuming the comparator price ${p}_{B}$ is fixed, the implied value-based price for D follows from Eq. (18). In this setting, post-trial extrapolation primarily concerns hospitalization-related cost offsets rather than survival costs. If the shortcut’s structural stability assumptions are plausible—namely, a stable mapping from avoided events to health benefits and stable unit event costs—then extending the horizon is not expected to materially change the implied price.

We use representative values (illustrative and not jurisdiction-specific) to show how a trial-period value-based price can be obtained (Table 6). We set the trial-period ICER equal to $\lambda$:

$$\lambda \hspace{0.25em} =\hspace{0.25em} \frac{p\cdot T\hspace{0.25em} -\hspace{0.25em} {Savings}_{trial}}{\Delta {E}_{trial}}$$

- QALYs gained: $$0.04\times 3=0.12$$.
- Hospitalizations avoided: $$0.15\times 0.15\times 3=0.0675$$.
- Savings: $$0.0675\times \mathrm{10,000}={\in} 675$$.

Solve for $$p$$:

$$\mathrm{50,000}=\frac{3p-675}{0.12}\hspace{0.25em} \Rightarrow \hspace{0.25em} \mathrm{6,000}=3p-675\hspace{0.25em} \Rightarrow \hspace{0.25em} p={\in}{2,225}/year$$

In a conventional lifetime model, discounted costs and QALYs are summed over time, where $t$ indexes time periods (e.g., years). If survival is identical across arms (as suggested by the absence of a mortality effect in PARAGON-HF), the survival probability $S(t)$ enters both the numerator and denominator symmetrically:

$$ICE{R}_{lifetime}=\frac{\sum_{t}S(t)\hspace{0.17em}(\Delta p+\Delta {C}_{nonprice}-{Savings}_{t})}{\sum_{t}S(t)\hspace{0.17em}\Delta {E}_{t}}$$

When $\Delta {E}_{t}$ and ${Savings}_{t}$ are stable per period (or change proportionally over time), the common survival weighting cancels, leaving the incremental cost–effect ratio determined by the per-period increments rather than by the chosen horizon. Hence, under the shortcut assumptions, the value-based price that satisfies the threshold over the trial horizon is the same price that would satisfy it over a longer horizon.

This illustration shows that when a treatment reduces morbidity (e.g., hospitalizations) without changing survival, complex lifetime extrapolation may be non-decision-relevant for the implied value-based price—provided the shortcut’s structural stability assumptions are judged plausible.

### Empirical evidence for underlying conditions

In this section, we discuss the applicability of the proposed shortcuts based on the availability of empirical evidence supporting their underlying assumptions. Consistent with Proposition 1, some interventions can be assessed using a single focal CE in the analysis—for example, clopidogrel or aspirin when the modeled endpoint is restricted to reinfarction in patients with a history of myocardial infarction; vaccines when focusing on preventing a specific infection outcome; and life-saving medications such as antibiotics when the analysis focuses on preventing death from a specific infection.

For multiple CEs, an underlying assumption of Propositions 2 to 7 is that interventions prevent death solely by preventing CEs, not by reducing the probability of dying from a CE once it occurs. This assumption is evident in treatments like those for osteoporotic fractures, which prevent death by avoiding fractures rather than reducing fatality rates post-fracture. Conversely, aspirin not only prevents myocardial infarction but also reduces the likelihood of dying from such an event, illustrating a scenario where the additional assumption—that disease-specific and unrelated expenditures increase concurrently with age—becomes relevant.

For cases involving multiple CEs, the shortcuts may require maintaining approximately constant ratios of CE costs, fatality rates, and severity weights across endpoints. This includes scenarios where the cost, death rate, or severity of each CE does not change with age. From our review of the literature, this latter assumption is common in modeling studies, often due to the limited availability of age-related data.

Propositions requiring a constant ratio of incidence rates of CEs are particularly applicable when these rates increase uniformly over a lifetime. This pattern has been observed in cardiovascular diseases such as coronary heart disease and strokes. Studies like the Honolulu Heart Program [21] and the Framingham Offspring Study [30] have used age as an untransformed explanatory variable in multivariate regression analyses to predict the incidence of these conditions. When the estimated age effect is similar across endpoints (or is assumed to be similar), incidence rates can be treated as increasing proportionally with age, consistent with the proposition’s constant-ratio condition. In practice, this is often approximated in applications that model a composite endpoint and include age (transformed or untransformed) as a predictor—for example, the New Zealand Cardiovascular Risk Charts and the Joint British Societies Cardiovascular Risk Assessor. Such composite-endpoint models capture the age gradient of overall risk but typically do not identify component-specific age trends; thus, heterogeneous age profiles across individual CEs could in principle violate the constant-ratio assumption even when the composite appears stable. In practice, large short-run reversals in component trends are uncommon, so proportionality can be a reasonable simplification. Note that the assumption that incidence rates of CEs increase uniformly with age also accommodates scenarios where these rates increase non-linearly or exponentially. For example, in the case of osteoporotic fractures, the incidence of both hip and vertebral fractures in men has been shown to increase exponentially with age [8].

When applying these shortcuts to an analysis that uses QALYs instead of CEs, the same structural logic applies after mapping each clinical event (CE) into an associated QALY loss. A linear mapping between events avoided and QALYs gained is defensible locally—at a given age and comorbidity level—and it can also remain valid over extended horizons if post-trial changes in morbidity and quality of life affect all relevant endpoints in a common, proportional manner. In practical terms, this requires that (i) the post-event utility profile for each CE (acute decrement, recovery pattern, and any long-run decrement) is stable over time or changes proportionally across CE types, and (ii) baseline utility and comorbidity evolution does not introduce differential non-linearities across endpoints or across comparisons. Under such “common-scaling” conditions—such as when a composite endpoint is affected uniformly—non-linear age- or comorbidity-dependence in QALY decrements does not necessarily alter the implied value-based price because it enters symmetrically in both treatment comparisons (i.e., on both sides of the pricing condition) and therefore preserves the proportionality relationships on which the shortcuts rely.

Consistent with the CE-based propositions, the assumptions further require that mortality patterns linked to each CE evolve in a stable way: either CE-associated mortality increases proportionally with general population mortality (i.e., a stable relative excess mortality) or it remains approximately constant. For example, long-run mortality after stroke has been reported to remain at an approximately constant multiple of general population mortality among longer-term survivors [15]. As an example of the latter assumption, some models assume approximately constant post-onset mortality rates within relevant strata following dialysis initiation.

These conditions are most likely to be violated in settings with heterogeneous endpoints and evolving health states—such as oncology (progression, subsequent-line therapies, and time-varying quality of life), multimorbidity with recurrent events and interaction effects, or diseases with substantial recovery/rehabilitation trajectories—where QALY decrements can vary non-linearly with age, baseline utility, comorbidity, and time since event in ways that differ across endpoints (e.g., step changes at progression and transient but severe decrements from acute grade 3–4 toxicities). In such cases, the mapping from events avoided to QALYs gained may no longer preserve the proportionality conditions, and long-term utility modeling becomes more likely to be decision-critical for pricing.

This section provides illustrative support for the plausibility of the proposition assumptions by citing common modeling conventions and broad epidemiological patterns. However, we do not formally test whether these assumptions hold in a given disease area or whether departures from them would materially alter value-based pricing decisions. More generally, the robustness of shortcut-based pricing to violations of the underlying assumptions is necessarily context-specific: whether a given deviation is “acceptable” depends on the decision-maker’s tolerance for pricing error and on the magnitude of post-trial components relative to trial-period effects and costs. Accordingly, no single simulation can establish robustness universally across propositions and applications. Therefore, the shortcuts should be applied only after assessing empirical support for the relevant assumptions and examining robustness to plausible violations.

## Discussion

This paper identifies conditions under which complex decision-analytic modeling (e.g., Markov models or discrete-event simulation) is unnecessary beyond the trial period for VBP. These conditions require that at least one comparator has been assessed under VBP. In therapeutic areas with sequential entrants, such evaluations are increasingly common. In relation to the present paper, the shortcuts are derived for a given pricing context (i.e., taking comparator prices/net prices and the relevant threshold as fixed at the decision point). They can therefore be applied at any appraisal or reassessment time point, but do not remove the need to consider life-cycle price changes or sequential entry when those dynamics are central to the decision problem [14, 31]. In applying the shortcuts, modelers should verify that the empirical setting plausibly satisfies the required conditions (e.g., proportionality and stability assumptions). When specific conditions are met, they eliminate the need to calculate health benefits, such as QALYs, beyond the clinical trial period for VBP purposes. However, it remains necessary to calculate health benefits within the trial period. Depending on which assumptions are satisfied, it is sometimes possible to completely avoid modeling savings and life-extension costs beyond the trial period. In other cases, it may only be possible to avoid modeling either savings or life-extension costs, but not both.

When modelers have previously used a decision model for the comparator treatment, they may employ the same model to determine the value-based price of the new compound. In this scenario, the shortcuts facilitate the validation of the calculated price. If model assumptions have evolved due to changes in input parameters, modelers might still conduct a validation check using the initial model’s parameters, assessing the VBP of the new drug based on these parameters and comparing the result obtained from applying the shortcut.

There are cases where the manufacturer of a comparator drug or a new drug sets a price below the value-based price to capture a larger market share, or the comparator drug has become generic. In these cases, the ICERs of the comparator drug and the new drug are not the same. However, this discrepancy does not limit the applicability of the proposed shortcuts. For expositional purposes, the price gap can be represented as an additive constant in the comparator’s drug-cost term (i.e., a time-invariant level shift). Because this constant is the same in the trial-period and post-trial expressions, it cancels when deriving the price change for the new drug induced by extending the time horizon. This holds true because the shortcuts address changes in the price of the new drug when extrapolating costs and health benefits beyond the trial period. Similarly, a change in the maximum willingness to pay over time, leading to a higher acceptable ICER for the new drug compared to the comparator, does not affect the proposed shortcuts. This change is already reflected in the value-based price set in the trial-period cost-effectiveness analysis (i.e., the within-trial ‘piggyback’ evaluation or a decision model calibrated to the trial period) using the prevailing willingness-to-pay threshold.

Outside the realm of VBP, these shortcuts can still be informative in two related ways. First, even when comparator prices are not set by VBP, the shortcuts can be used as a screening tool: they help assess whether extending the analysis beyond the trial horizon would materially change the trial-based price signal at a chosen (or implied) threshold; if not, complex long-term modeling is unlikely to be decision-critical for pricing. In this sense, they apply the pricing logic underlying Eq. (18) locally, using the observed comparator price as a fixed benchmark rather than requiring that it be value-based. Second, the shortcuts can be applicable in scenarios where trial-based analysis indicates extended dominance. In such cases, a new drug is more effective and also has a lower ICER compared to its comparator [22]. The reason why these shortcuts are suitable in cases of extended dominance, but not strict dominance, is that a simple comparison between two interventions—sufficient to establish strict dominance—is inadequate for applying these shortcuts. If the conditions for a shortcut are met, long-term modeling is not necessary for the decision conclusion. Where a long-term model is nonetheless required, the shortcut can be used as an internal consistency check.

Our results speak directly to the methodological literature on extrapolation and structural uncertainty in HTA. Extrapolation beyond trial follow-up is commonly required to estimate long-term benefits and costs, particularly for survival, yet different plausible modeling choices can yield materially different ICERs—making extrapolation both consequential and resource intensive [17, 18]. More broadly, recent guidance and reviews emphasize that structural uncertainty (e.g., choice of model structure, extrapolation approach, and how outcomes and costs are linked) should be addressed transparently and explored through scenario/structural sensitivity analyses, rather than being treated as secondary to parameter uncertainty [5]. This paper complements that literature by shifting the focus from how to extrapolate to when extrapolation is decision-relevant for value-based pricing: we identify sufficient conditions under which adding post-trial components does not change the implied value-based price, and conditions under which only limited components require projection. In doing so, the proofs can be interpreted as delineating a class of settings in which structural uncertainty from long-term extrapolation is (for pricing purposes) theoretically “non-binding,” versus settings in which extrapolation choices are likely to change conclusions.

The implications also relate to long-standing reference-case discussions about which cost categories should be included in CEA and how to transparently report consequences. The Second Panel on Cost-Effectiveness in Health and Medicine emphasizes clarity about included consequences (e.g., via an impact inventory) and careful interpretation of results under defined analytic conventions [26]. Our conditions specify when certain downstream components—such as savings from avoided events and costs incurred in added life-years—do not affect the price implied by a threshold-based decision rule. This provides a formal justification for omitting or simplifying specific categories in restricted circumstances while maintaining transparency about what has been assumed.

This study is methodological and derives sufficient conditions analytically; therefore, the applicability of the shortcuts depends on whether the underlying assumptions are plausible in a given context. The shortcuts do not eliminate uncertainty in trial-based inputs (e.g., event rates, relative effects, costs, and weights), and standard uncertainty and sensitivity analysis remains necessary for any trial-period economic evaluation. The main methodological limitation is that several propositions rely on structural stability or proportionality assumptions (e.g., stable relative treatment effects across endpoints and over time, stable fatality proportions, and stable relationships between event incidence and costs). When these assumptions do not hold—such as with effect waning, treatment-effect heterogeneity, changing standards of care, age-dependent event patterns, or non-stationary costs (e.g., changing hospitalization practice or unit costs)—extrapolation and/or more detailed modeling may be required.

Importantly, the assumption of stable or proportionally changing relative treatment effects over time is frequently among the most contested elements in HTA. Consequently, long-term treatment-effect behaviour is often uncertain and routinely examined through alternative extrapolation scenarios (including, where plausible, time-varying effects or effect waning) rather than assumed to remain stable. Empirical evidence also indicates that proportional-hazards–type behaviour does not hold uniformly across therapeutic areas. For example, proportional hazards commonly fail in immuno-oncology and other oncology contexts, where delayed treatment effects, time-varying hazard ratios, and long-term “tail” survival have been widely documented [1]. Conversely, in some chronic secondary-prevention settings with sustained mechanisms of action and long-term follow-up (e.g., lipid-lowering therapies), meta-analytic evidence can support relatively stable relative effects over time [7], making proportionality assumptions more defensible as a first approximation.

In particular, the shortcuts should be applied with substantial caution, or not at all, in therapeutic areas characterized by complex and heterogeneous endpoints—such as oncology or multimorbidity—where equal or proportionally changing weights, costs, and mortality across events are unlikely to be credible, and where treatment effects may differ markedly across endpoints and over time. In such settings, long-term modeling is typically decision-critical because endpoint-specific trajectories, subsequent-line therapies, competing risks, and heterogeneous cost and quality-of-life impacts can materially affect the ICER and the implied value-based price.

Finally, because the paper focuses on internal verification and logical consistency, external validation is not performed. In practice, analysts should assess empirical support for the relevant assumptions and interpret the shortcuts as decision aids rather than universal replacements for long-term models.

## Conclusion

The purpose of this paper was to identify under what conditions savings and survival costs can be disregarded. Therefore, this paper has provided an example of how to minimize data and model requirements once a health care system has established a framework for VBP. This approach could serve as a model for other health care systems seeking ways to reduce the information needed to determine drug prices within their specific contexts. In practical application, analysts should verify whether empirical evidence supports the relevant proposition conditions before relying on a shortcut. As the shortcuts presented are based on the assumptions currently widely accepted, any future changes in these standard assumptions will necessitate an assessment of the transferability and adaptability of the shortcuts.

## Data Availability

No datasets were generated or analysed during the current study.
